# Comparative genomic insights into multidrug resistance in classical and hypervirulent *K. pneumoniae* clinical isolates

**DOI:** 10.1038/s41598-025-27122-6

**Published:** 2025-12-06

**Authors:** Azra, Ihsan Ullah, Woranich Hinthong, Jody E. Phelan, Susana Campino, Ihtisham Ul Haq, Zia Ud Din, Sajjad Ahmad, Gulab Fatima Rani, Aneeqa Naz, Otavio Cabral-Marques, Muhammad Zubair Bhatti, Taj Ali Khan, Taane G. Clark

**Affiliations:** 1 Institute of Pathology and Diagnostic Medicine, IP&DM, Khyber Medical University Peshawar, Peshawar, Pakistan; 2 Faculty of Infectious and Tropical Diseases, London School of Hygiene and Tropical Medicine, London, UK; 3 Post-Graduate Programme in Innovation in Technology, Federal University of Minas Gerais, Belo Horizonte, Minas Gerais 30150-240 Brazil; 4 Department of Pharmacy, Kohat University of Science and Technology, Kohat, Pakistan; 5 Department of Pathology, Multan Medical and Dental College, Multan, Pakistan; 6 Department of Immunology, Institute of Biomedical Sciences, University of São Paulo, São Paulo, 05508-220 Brazil; 7 Department of Medicine, Division of Molecular Medicine, Laboratory of Medical Investigation 29, University of São Paulo School of Medicine, São Paulo, Brazil; 8 Department of Clinical and Toxicological Analyses, School of Pharmaceutical Sciences, University of São Paulo, São Paulo, Brazil; 9 D’Or Institute for Research and Education, São Paulo, Brazil; 10 Lady Reading Hospital, Peshawar, Pakistan; 11 Public Health Reference Laboratory, Khyber Medical University, Peshawar, Pakistan; 12 Faculty of Epidemiology and Population Health, London, School of Hygiene and Tropical Medicine, London, UK

**Keywords:** *K pneumoniae*. hypervirulence, Resistance genes, Hybrid hvKp-MDR, Antimicrobial sequencing, WGS, Computational biology and bioinformatics, Genetics, Microbiology, Molecular biology, Diseases, Medical research

## Abstract

*Klebsiella pneumoniae* is a highly adaptable opportunistic pathogen responsible for various infections, particularly in immunocompromised individuals. The recent genetic evolution of *K. pneumoniae* has led to the emergence of strains exhibiting both hypervirulence and multidrug resistance (MDR). In this study, we compared the genomic characteristics of MDR in classical and hypervirulent *K. pneumoniae* clinical isolates. Sixty-four isolates were collected from two healthcare institutions in Khyber Pakhtunkhwa, Pakistan, and identified using standard microbiological techniques. Whole-genome sequencing (WGS) was performed on 30 selected isolates using an Illumina platform. The sequencing data were analysed with the Kleborate tool. WGS analysis identified sequence types ST147 (n = 7, 23%) and ST2629 (n = 5, 16.6%) as the most prevalent lineages. Additionally, 13 other ST were detected, including ST147-1LV (n = 1, 3.3%) and ST859 (n = 1, 3.3%), which exhibited hypervirulent MDR traits. Among K-loci-associated virulence determinants, KL64 was the most predominant (n = 7, 23%), while the O-serotype O1/O2v1 was found in 22 isolates. A diverse range of antimicrobial resistance (AMR) determinants was observed across isolates. Plasmid-mediated quinolone resistance (PMQR) genes (*qnr*S1, *qnr*B1, *qnr*B4, *qep*A2) and aminoglycoside-modifying enzymes (*aac*(3)-IIa, *aac*(6′)-Ib-cr, *strA*, *strB*) were detected in both classical and hypervirulent strains. β-lactamase genes included *bla-*SHV variants (*bla*-*SHV-1*, *bla*-*SHV-11*, *bla*-*SHV-25*, *bla*-*SHV-187*), *bla-CTX*-*M-15*, *bla*-*VEB-5*, *bla*-*OXA-1*, *bla-OXA-48*, and *bla*-*OXA*-*181*. Additional resistance genes conferred resistance to macrolides (*ermB, mphB*), phenicols (*catB4, catA1, floR*), sulfonamides (*sul1, sul2*), tetracyclines (*tetA, tetB, tetD*), and trimethoprim (*dfrA1, dfrA12, dfrA14*). Acquired AmpC and other β-lactamases (*DHA-1*, *TEM-1D*, *CMH-1*, *CMY-6*, *LAP-2*, *Amp*C1) were also present. The most prevalent plasmid replicons were Col(pHAD28), IncFIB(K), and IncR, with the hypervirulent ST147-1LV isolate carrying the highest number. These findings underscore the significant public health threat posed by hypervirulent and MDR *K. pneumoniae* strains, highlighting the extensive burden of virulence and resistance genes that complicate treatment strategies.

**Supplementary Information:**

The online version contains supplementary material available at 10.1038/s41598-025-27122-6.

## Introduction

*Klebsiella pneumoniae* is a highly pathogenic Gram-negative bacterium known for its rapid mutation rate, which facilitates the emergence of hypervirulent and multidrug-resistant (MDR) strains^1,2^. This relentless evolution has rendered many antimicrobials ineffective against *K. pneumoniae*^1^. The pathogen is categorised into two distinct pathotypes: hypervirulent *K. pneumoniae* (hvKp) and classical *K. pneumoniae* (cKp)^2^. These pathotypes differ molecularly, geographically, and clinically. The World Health Organization (WHO) has identified cKP isolates as a “critical concern” because they frequently cause nosocomial infections in immunocompromised individuals or those with underlying illnesses, and they have progressively gained antibiotic resistance determinants, primarily carbapenemases^3–5^. However, hvKP has become a virulent pathogen in recent decades that can infect healthy people with invasive community-acquired illnesses. Rapid metastatic spread and the development of pyogenic tissue abscesses are characteristics of these infections^5^.

cKp is predominantly associated with nosocomial infections, and hvKp is responsible for community-acquired infections^2^. In K. pneumoniae, both classical and hvKp strains exhibit significant AMR and virulence, complicating infection severity^2^. Classical strains are often linked to nosocomial infections and display multidrug resistance through mechanisms like extended-spectrum beta-lactamases (ESBLs)^2,6^. Hypervirulent strains possess aggressive pathogenic traits and carry antibiotic resistance profiles^2,7,8^. This combination of heightened virulence and robust resistance in hypervirulent strains makes them particularly challenging to treat^7,8^.

Classical and hypervirulent *K. pneumoniae* strains possess key virulence factors, such as capsular polysaccharides (CPS) and lipopolysaccharides (LPS), encoded by K-loci and O-loci, respectively. To date, 134 K-types and 11 O-antigens have been identified^9,10^. Both CPS and LPS can suppress early inflammatory responses, defend the bacteria against phagocytosis, opsonisation, and antimicrobial peptides, and stimulate the host’s innate immune response^11^. The heightened immunogenicity and prominent surface exposure of CPS and LPS in *K. pneumoniae* make them candidates for vaccine development^12^. CPS types KL1 and KL2 are linked to increased virulence, while KL47 and KL64 are associated with both hypervirulence and carbapenem resistance^13^. Diversity in capsular types has been identified, with most types associated with specific sequence types (STs); for example, ST11 with K27 or K64, ST101 with K17, ST340 with KL151, ST15 with K24, and ST17 with KL112^14^. The structural diversity of the O-antigen enhances immune evasion^15^. The plasmids often harbour genes essential for virulence and antibiotic resistance^16,17^. Genomic surveillance is increasingly used to monitor hypervirulence and carbapenemase genes in *K. pneumoniae*, particularly in high-risk clonal complexes like CC258 (ST11, ST258, ST512, and ST437)^1,18^. The emergence of hypervirulent and carbapenem-resistant strains (CR-hvKp) has become a significant global health threat^19–21^. CR-hvKp infections are challenging to treat with available medicines and warrant more investigation^19–21^.

Whole Genome Sequencing (WGS) has emerged as a critical tool for identifying and monitoring the genetic determinants of bacterial virulence and resistance, and enables a deeper understanding of the mechanisms underlying the pathogen’s virulence, resistance, and adaptability^2,4,6,21,22^. Carbapenem resistance in *K. pneumoniae* mainly results from carbapenemase production (e.g., KPC, NDM, OXA-48), often combined with porin loss and efflux pump activation, which together reduce antibiotic efficacy and lead to high-level resistance.^23,24^. This research aims to conduct a genomic analysis of classical and hypervirulent *K. pneumoniae* strains from Pakistan to elucidate the diversity of genes associated with virulence, antibiotic resistance, and plasmids across different sample sources.

## Materials and methods

### Sample collection and pathogen isolation

The study included 500 samples sourced from urine, pus, wounds, blood, and other body fluids collected from patients of Mardan Medical Complex (Mardan) and Ayub Medical Complex (Abbottabad), Khyber Pakhtunkhwa (KPK), Pakistan, to isolate *K. pneumoniae.* Informed consent was obtained from all participants, and ethical approval was granted. The samples were aseptically inoculated onto sterile Blood and MacConkey agar (Oxoid, UK) medium and incubated at 37 °C for 24 h. *K. pneumoniae* were identified by biochemical profiling, and strain confirmation was determined using the API 20E kit (bioMérieux SA, Lyon, France).

### Genomic DNA isolation and whole genome sequencing

*K. pneumoniae* preserved isolates were meticulously inoculated onto Mueller–Hinton Agar plates and incubated for 24 h at 37 °C. DNA extraction was performed using the DNeasy UltraClean microbial kit (Qiagen, Germantown, MD, USA). DNA quantification was performed using the Qubit dsDNA fluorometer (ThermoFisher Scientific, Waltham, MA, USA). Based on the phenotypic susceptibility profiles, thirty samples with high-quality DNA underwent WGS at The Applied Genomics Centre, London School of Hygiene and Tropical Medicine. Before library preparation and sequencing, DNA quantity and quality were re-evaluated. Libraries of bacterial genomes were prepared using the Illumina library preparation kit (San Diego, CA, USA), following the manufacturer’s protocol. Sequencing was conducted using the Illumina MiSeq platform, generating paired-end short reads with an average length of 150 bp and approximately 50-fold coverage.

### Data processing and quality control

The FastQC (v0.11.9) tool was employed to conduct comprehensive pre-processing of FastQ files^25^, and Trimmomatic (v0.36) software was used to remove adapter sequences^26^. Data-quality reports were generated pre- and post-trimming and then consolidated into a single report using MultiQC. Before genome assembly, paired-end reads underwent taxonomic classification with Kraken2 software^27^, employing default parameters to determine species identity and assess potential contamination. Kraken2 outputs were visualised using a Krona chart, providing a graphical overview for contamination assessment. Processed paired-end reads were then assembled de novo using the SPAdes genome assembler^28^ within the Shovill pipeline (https://github.com/tseemann/shovill; accessed on 20 May 2024) with default settings. Assembly statistics, such as N50 values and the number of contigs, were calculated with QUAST using default settings^29^.

### Genomic profiling

The de novo assembled sequences served as the basis for strain characterisation. Species verification was cross-referenced using the online Type Strain Genome Server (TYGS; https://tygs.dsmz.de; accessed on 15 June 2024). To investigate antibiotic resistance genes and the presence of mobile genetic elements, the web tools ResFinder (v4.1) and PlasmidFinder (v1.0 and v2.1) were employed^30,31^. Serotyping, cgMLST and cSNP-phylogenetic analyses were conducted using the online Pathogenwatch platform (https://pathogen.watch; accessed on 10 June 2024). The MLST scheme from PubMLST was utilised to assign a sequence type (ST) to each strain. For the validation of AMR detection, the ResFinder database was applied for alignment-based analysis. Assembled genomes were aligned against the database using BLASTn, with only alignments exhibiting more than 90% identity and over 60% target coverage being retained. Genes related to virulence were detected using ABRicate (https://github.com/tseemann/abricate; accessed on 3 June 2024) using the Virulence Factor Database (VFDB)^32^. Along with species identification, we used Kleborate software to methodically evaluate the existence of virulence determinants and resistance, as well as STs and virulence scores^33^.

### Genome annotation and phylogenetics 

Genome annotation was conducted using Prokka software, which delineated features such as coding sequences (CDS) and ribosomal and transfer RNA genes. Various standard output files were generated for subsequent analyses. The Roary pipeline utilised Prokka’s GFF-generated files to construct pan-genomes and perform core-genome-based phylogenetic analysis^34^. The core genome was defined based on the criteria of 95% identity for protein matches and the presence in 99% of strains. A multi-fasta alignment of all isolates was performed to construct a phylogenetic tree using IQ-TREE with default parameters. Subsequently, a core genome-based phylogeny was constructed to facilitate the clustering of all isolates and visualised in the iTOL^35^. Variant calling and filtering were performed using the Snippy pipeline (v4.6.0), leading to a SNP-based phylogeny.

## Results

### Genomic analysis and assembly statistics of *K. pneumoniae* strains

A total of sixty-four *K. pneumoniae*-positive samples were analysed. DNA extraction was performed, and thirty samples based on phenotypic profile and high DNA quality were selected for further processing (Suppl. Table 1, Suppl. Table 2). The WGS of *K. pneumoniae* were performed using the Illumina MiSeq platform. Assembly statistics of all the genomes are detailed (Suppl. Table 3). The genomes exhibited between 5,192 and 11,192 protein-coding sequences (CDS), 3 to 23 ribosomal RNA genes (rRNAs), and 72 to 149 transfer RNA genes (tRNAs). Their sizes ranged from 5,332,556 to 11,152,258 bp, with GC contents varying from 53.99% to 57.44%. Assembly quality metrics showed N50 values between 21,500 and 45,000 bp, with corresponding L50 values of 25 to 60, indicating reliable assembly continuity. Sequencing depth ranged from 50- to 150-fold, ensuring high-quality base coverage, and annotation revealed plasmid sequences and mobile genetic elements in several isolates. (Suppl. Table 3).

### Identification of high-risk sequence types and novel k-serotypes

**Fig. 1:**
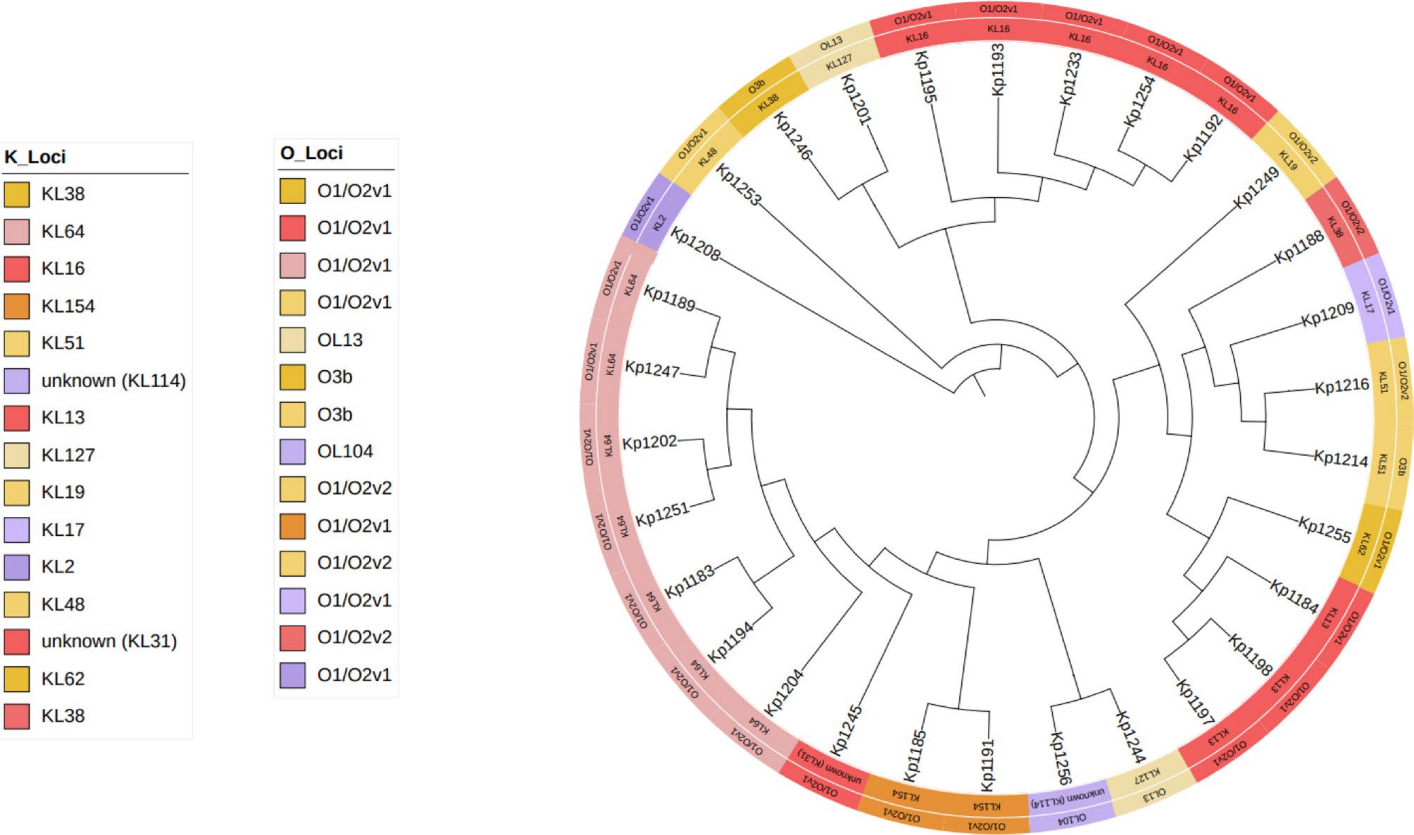
K loci and O loci distribution in the 30 isolates.

The isolates exhibited significant genetic diversity, with 15 distinct STs identified. Among these, high-risk STs such as ST147, ST2629, and ST4 were noted, alongside other types including ST147-1LV, ST1310, and ST37 (Fig. 1, and Suppl. Table 4). The study identified 14 K-serotype groups that characterise the *K. pneumoniae* population, primarily associated with the K loci. Observed capsule types included KL64 (n = 7), KL16 (n = 5), and KL114 (n = 1).

**Fig. 2:**
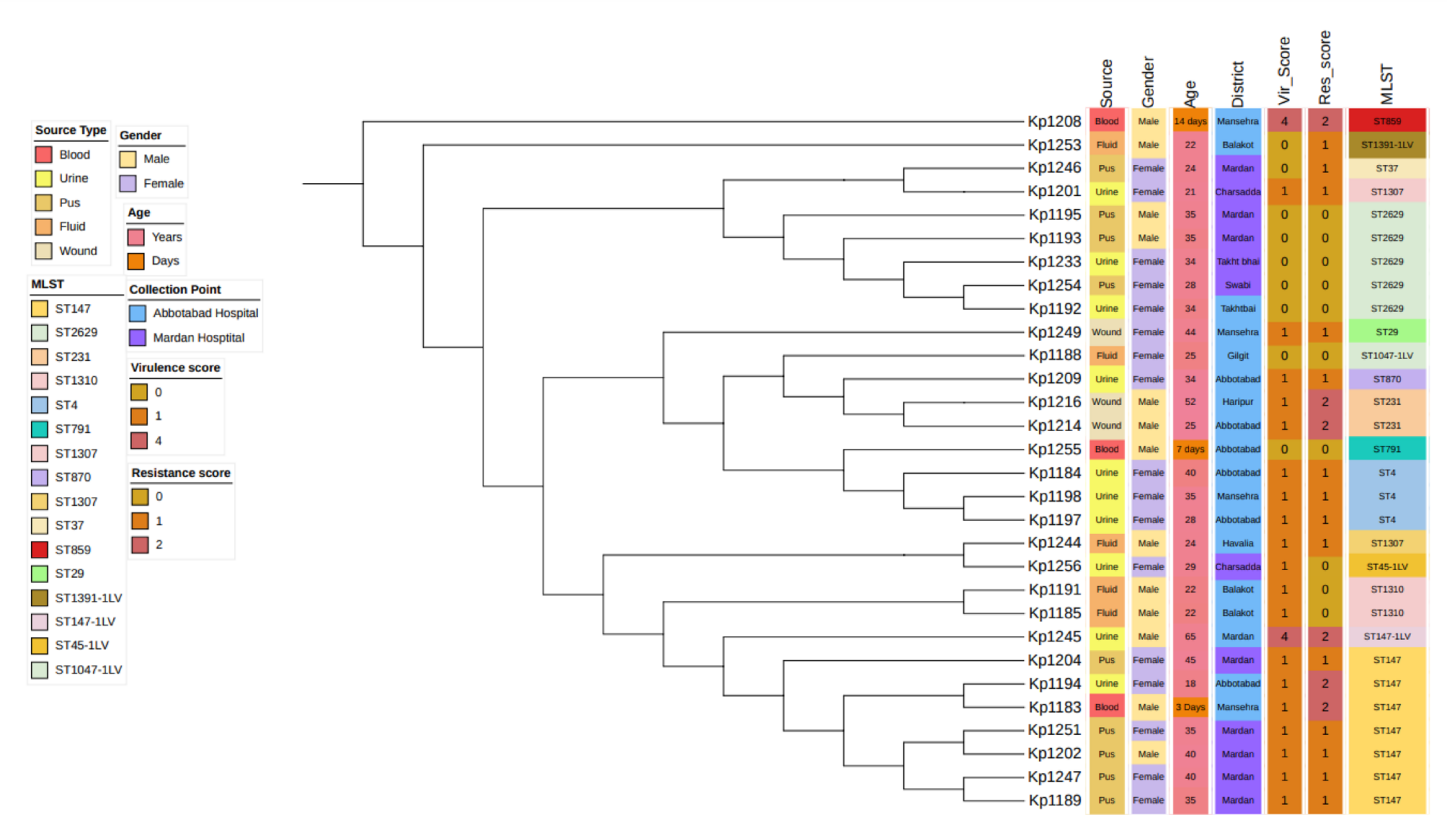
Phylogenetic tree based on core genes from 30 *K. pneumoniae* isolates, annotated with isolation source, gender, age, district, MLST, virulence score, resistance score, K loci, and O loci, generated by iTOL^35^.

Capsule types KL17, 19, 51, 64, 114, 127, and 151 are associated with hypervirulence, whereas KL2 and 31 are indicative of high-risk MDR clones, and KL13,16, 48, and 62 are characteristic of classical *K. pneumoniae*. Several isolates belonged to K serotypes where the serological capsule groups could not be precisely identified. Among the eight O-serotype groups identified, the predominant serotypes were O1/O2v1 (n = 22) and O1/O2v2 (n = 3). The remaining serotypes O3b (n = 2), OL13 (n = 2), O5 (n = 3), and OL104 (n = 1) were found at lower frequencies within the *K. pneumoniae* isolate population (Fig. 2).

### Identification of different AMR patterns

**Fig. 3:**
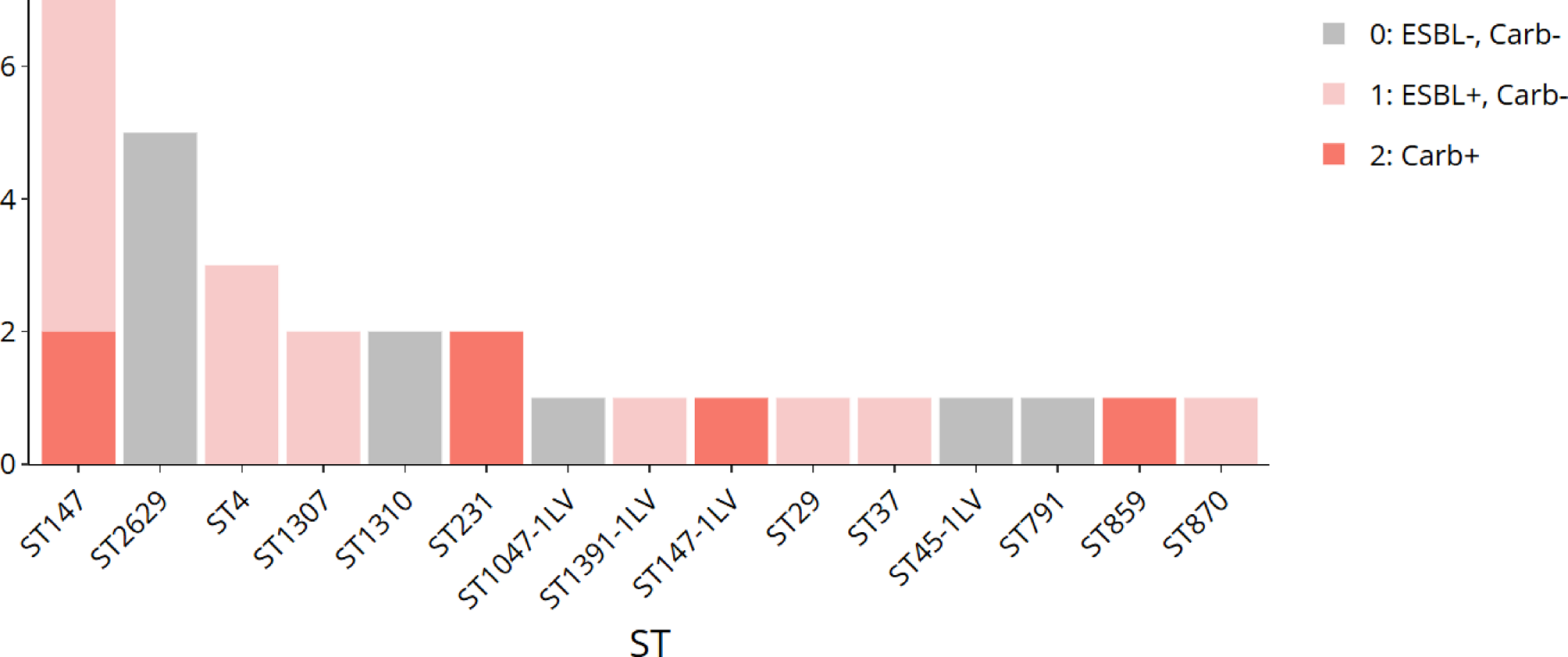
Isolate STs and resistance profiles as determined by Kleborate software^33^.

Antibiotic resistance genes were identified across nearly all isolates. Every strain exhibited resistance to SHV beta-lactamase; however, some did not exhibit changes at position 35Q. A diverse array of carbapenemase genes were detected, including *bla-NDM-1* (n = 1, 3.3%), *bla-NDM-5* (n = 2, 6.6%), along with other lactamases like *bla-OXA-1* (n = 3, 9.9%), and *bla-CTX-M-15* (63.3%). The ST859 harboured most of these carbapenemase genes, with a significant presence also in ST147-1LV, ST870, and ST1047. The high-risk clone ST859 harboured a broad repertoire of acquired and chromosomal resistance genes, including *CMH-1, CMY-6,* and *TEM-1D.v1,* and *SHV-11* (Suppl. Table 5). The ten strains lacking ESBLs comprised five of ST2629, one of ST1047-1LV, two of ST1310, one of ST791, and one of ST45-1LV (Fig. 3). Strains devoid of *bla-TEM-1D* included two of ST147, two of ST1310, five of ST2629, one of ST791, and one ST147-1LV (Fig. 3).

**Fig. 4:**
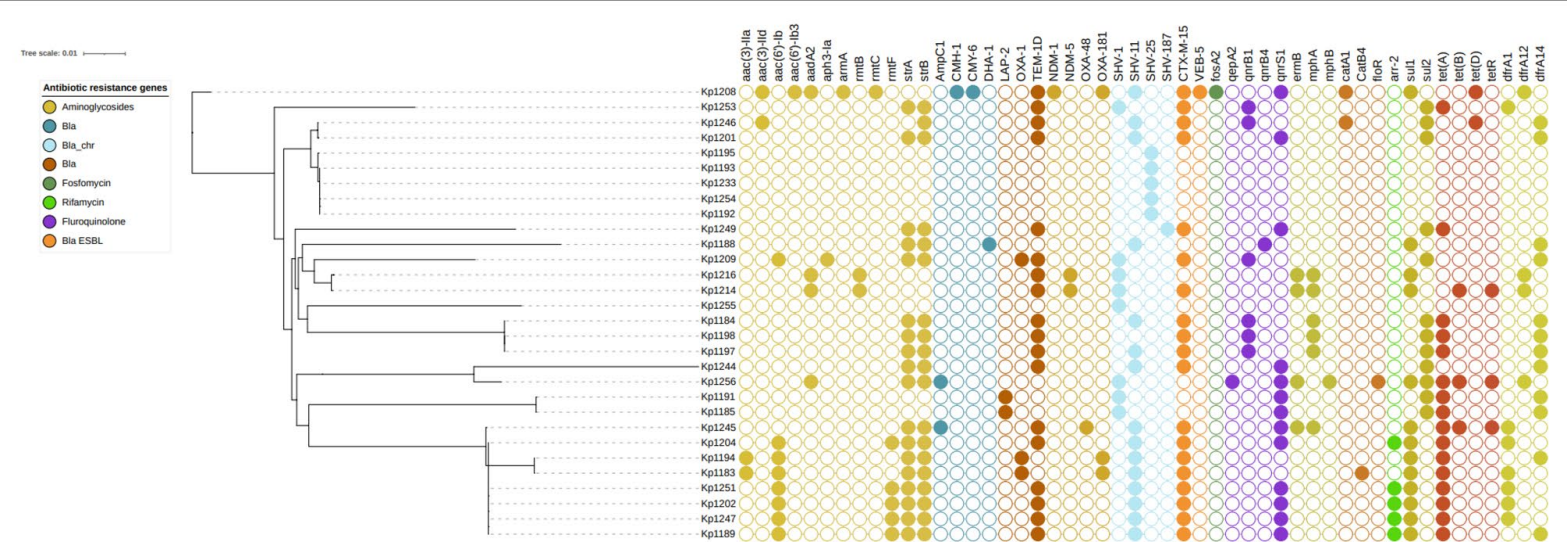
Core gene-based phylogenetic tree of 30 *K. pneumoniae* isolates with their corresponding resistance gene profiles. Filled circles indicate gene presence, while empty circles denote absence, as visualised in iTOL^35^.

Nine strains harboured more than three aminoglycoside resistance genes. Interestingly, twenty isolates exhibited phenotypic susceptibility to aminoglycosides despite the presence of corresponding resistance genes (Suppl. Table 6). The ST859 strain uniquely carried the *armA* gene. Among the 30 isolates, only one harboured the aminoglycoside phosphotransferase gene *aph3*. The most prevalent aminoglycoside resistance determinant was the plasmid-encoded streptomycin resistance gene B, *strB* (n = 19, 63%). Other identified resistance genes included acetyltransferase *aac*^3^-IIa *aac(6')-Ib, strA.v1,* and *aadA2*. (Fig. 4, Suppl. Table 5). Eighteen strains possessed a single gene, while two exhibited two genes linked to fluoroquinolone resistance. The most prevalent gene, *flq qnrS1*, was detected in 14 strains, including two in ST1310, two in ST1047-1LV, and six strains harboured the *flq qnrB1* gene. Twelve strains possessed the *Slusul1* gene, whereas 13 strains carried the *Slusul2* gene (Fig. 4, Suppl. Table 5). Nineteen strains were found to harbour genes conferring resistance to tetracyclines. Among these, 16 strains possessed the *tet(A)* gene, two strains carried *tet(D)*, and one strain contained *tet(B).v2*. Each resistant strain was associated with a single resistance gene. The *tet(D)* gene was present in ST859 and ST37, while ST231 harboured *tet(B)* (Fig. 4, Suppl. Table 5). The *drfA12* gene was found in four strains: one ST859 strain, two ST231 strains, and one ST45-1LV strain. Strains ST2629, ST29, and ST791 were negative for trimethoprim resistance genes (Fig. 4, Suppl. Table 5). *Oxa-48* and *Oxa-181* were detected in different STs.

#### Demonstration of rare resistance by some STs

Among the analysed strains, seven exhibited macrolide resistance, with four carrying the *ermB* gene and three harbouring the *mphA* gene, all within the ST4 lineage. Notably, only five of the ST147 strains possessed the *arr-2* gene, indicating rifampicin resistance, while the remaining strains were free from rifampicin resistance. The *fosA2* gene, associated with fosfomycin resistance, was found exclusively in ST859. Comprehensive resistome and phenotypic antibiogram data for all strains are presented (Fig. 4, Suppl. Table 5, Suppl. Table 6).

### Comprehensive analysis of enzyme alterations and genetic mutations

Mutations were observed in the Sulfhydryl Variable (SHV-11, SHV-25) enzyme. Mutations in *OmpK35* were detected in 7% of ST147 strains and 30% of ST231 strains. Concurrently, a mutation in *OmpK*36 resulted in aspartic acid (D) replacing glycine (G) in all ST231 strains (Suppl. Table 5). However, the ST147-1LV strain exhibited multiple mutations in *gyrA*, such as the replacement of serine (S) with isoleucine (I) and leucine (L) at position 83 and aspartic acid (D) with asparagine (N) at position 87 (Suppl. Table 5). A truncated, unaltered *CTX-M-15* gene was found in ST231, while the *strA* (streptomycin resistance gene) was identified in ST37 (Suppl. Table 5).

### Prevalence of virulence-associated factors

**Fig. 5:**
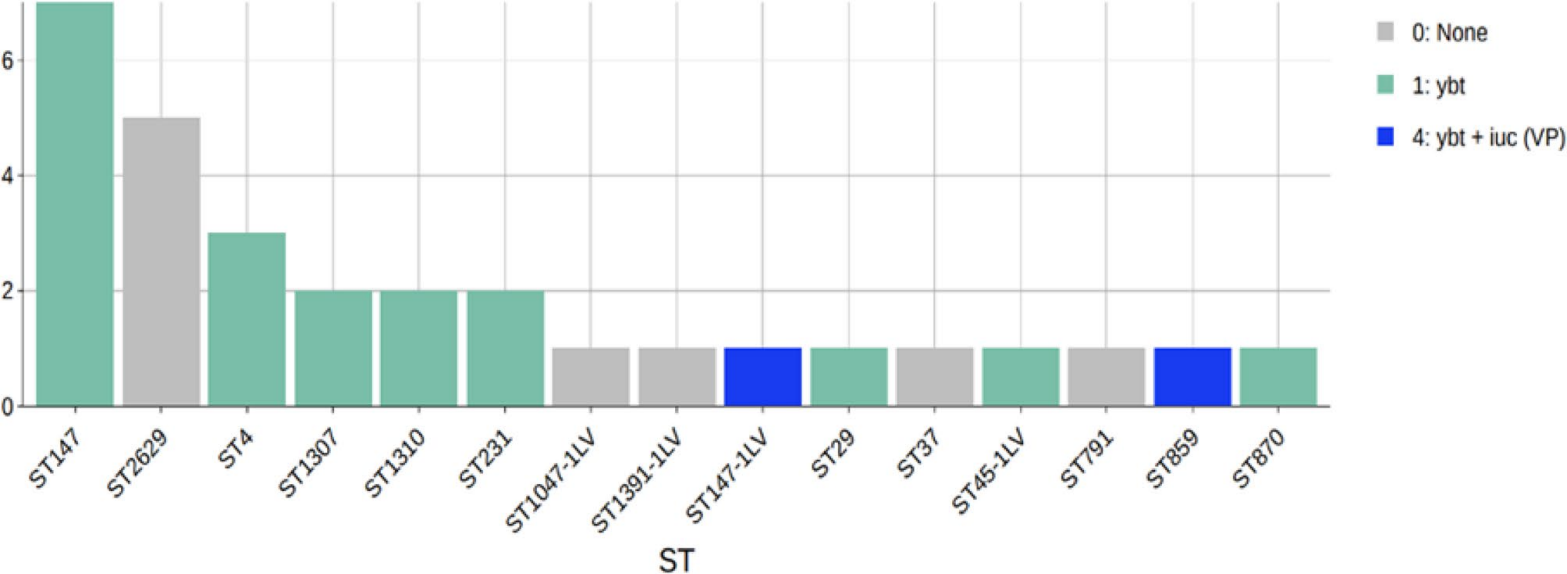
Isolate STs and virulence scores as determined by Kleborate software^33^.

Six strains tested positive in the string test, indicating the presence of multiple hypervirulent strains. Yersiniabactin emerged as the most prevalent virulence factor, found in 22 isolates. The most common variant was *ybt16*, associated with *ICEKp12* and identified in 8 isolates, particularly in ST147 strains (with one strain carrying a truncated gene), as well as in ST859. Three isolates harboured *ybt10* linked to *ICEKp4*, found in ST1310 strains and ST45-1LV. An incomplete *ybt9* associated with *ICEKp3* was present in ST147-1LV. Only two strains contained Aerobactin genes: ST859 carried *iuc1*, and ST147-1LV harboured *iuc5*. Neither colibactin nor salmochelin was detected in any isolates (Figs. 2 and 5, and Suppl. Table 4). Hyper-mucoviscosity, governed by the regulator of mucoid phenotype A (*rmpA*) and the transcriptional activator (*rmpA2*) genes, is closely associated with hypervirulence^2^.

### Virulence and resistance score

This study detected a maximum of 67 virulent genes across the isolates. Notably, the acquired virulence genes coding for colibactin, nutrition factors, salmochelin, and rmpA were absent. Yersiniabactin, an iron uptake locus (ybtAEPQSTUX), was identified in 21 (70%) isolates, while the regulatory gene *rmpA2* was present in only one isolate. Aerobactin, a dominant siderophore, was found in two (6.6%) isolates belonging to ST859 and ST147-1LV. Isolates with a resistance score of 0 belonged to ST1310, ST1047, ST2629, ST791, and ST45-1LV. The AMR score was calculated based on the presence of ESBL (1 score), carbapenemase without colistin resistance (2 scores), and carbapenemase plus colistin resistance (3 scores), with all other cases scoring zero (Figs. 2 and 5, Suppl. Table 4, Suppl. Table 5).

### Prevalence and distribution of plasmid replicon families

**Fig. 6:**
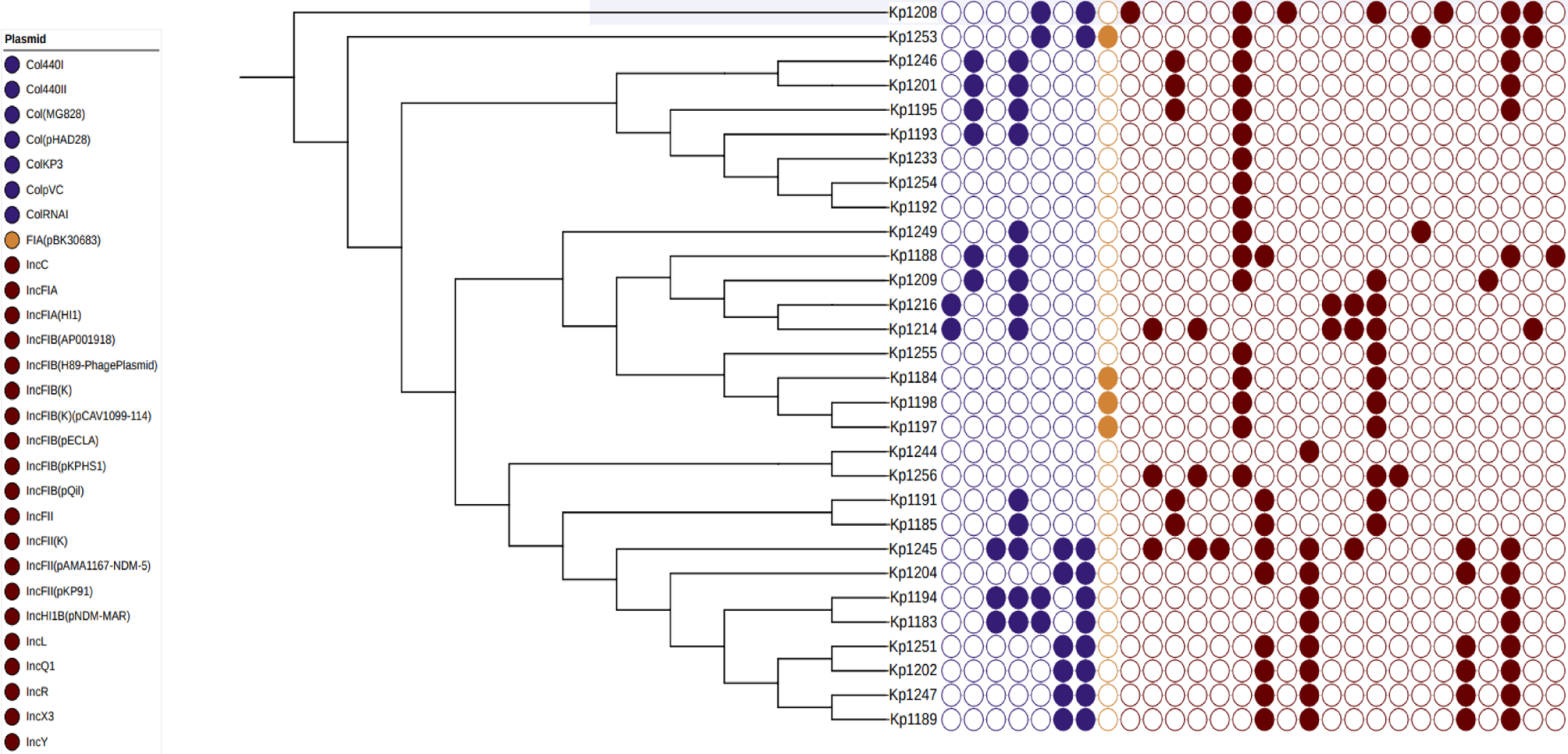
Core genome-based Phylogenetic tree of 30 *K. pneumoniae* isolates with their plasmid replicons. Empty shapes show the absence of a plasmid; filled shapes show the presence of a plasmid, generated by iTOL^35^.

Among the 28 replicon families identified by PlasmidFinder software, three were present in more than 45% of the isolates: IncFIB(K) in 57%, Col(pHAD28) in 50%, and IncR in 47%. Notably, ST147-1LV exhibited the highest number of replicons^16^, followed by ST231 with eleven and ST859 with nine replicons. On average, each isolate contained five replicons. Specifically, three ST2629 and one ST1307 isolates each harboured a single replicon (Fig. 6, Suppl. Table 6).

### Phylogenetic analysis of *K. pneumoniae* isolates

**Fig. 7:**
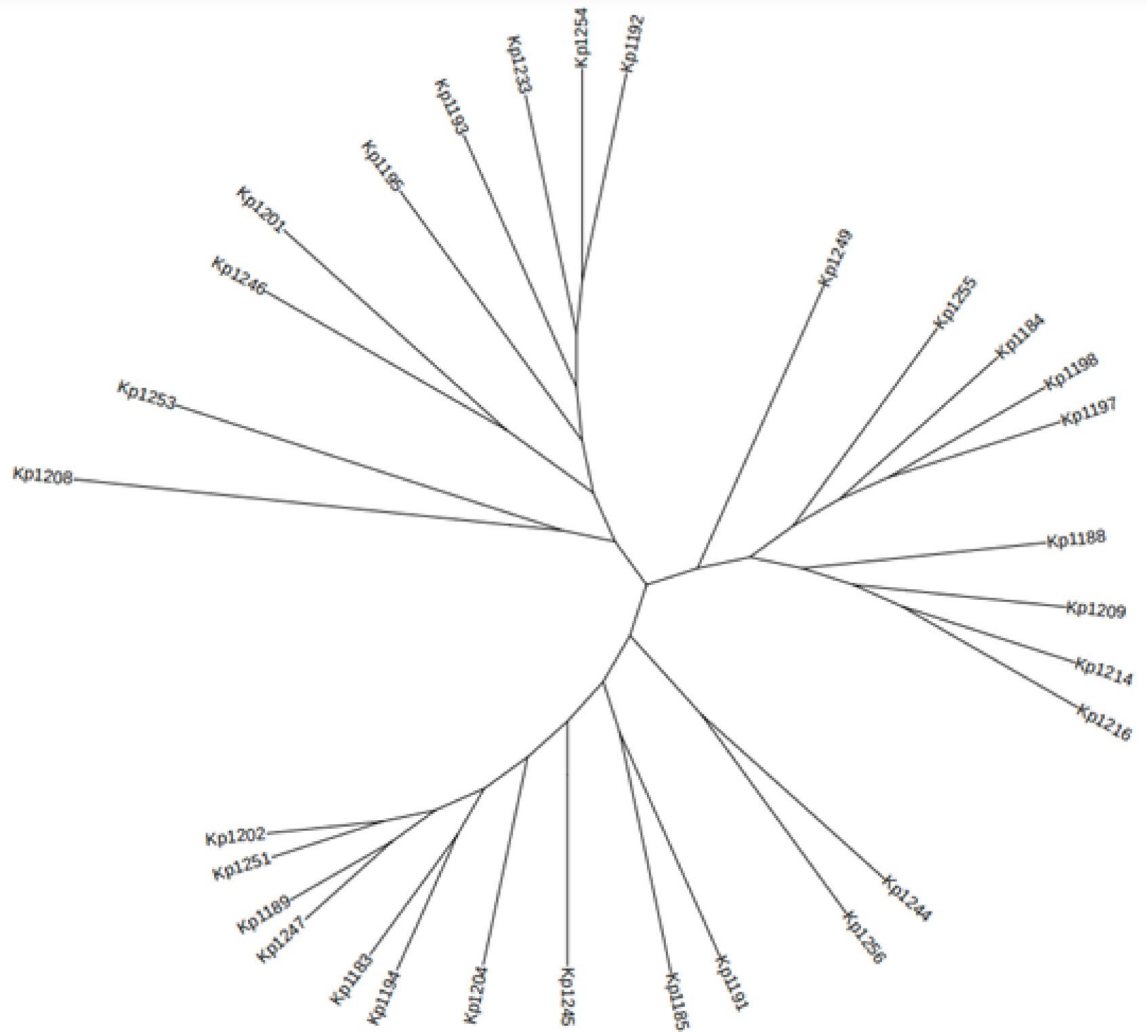
Core genome SNP-based phylogenetic tree of *K. pneumoniae* isolates, generated with FigTree.

The phylogenetic analysis of *K. pneumoniae* isolates (Fig. 7), revealed substantial genetic diversity. The core genome of the Klebsiella dataset comprises 3,935 genes. Four distinct clusters were identified from the analysed genomes. The ST859 and ST1391-1LV strains formed a unique cluster, distinguishing them from all other strains. Another cluster included ST37, ST1307, and ST2629. A third cluster comprised ST791, ST4, and ST29, along with isolates of ST231, ST870, and ST1047-1LV. The final cluster, the largest phylogroup, comprised six groups formed by the remaining 12 isolates, including sequence types ST147, ST147-1LV, ST1307, and ST45-1LV.

## Discussion

Globally, AMR is regarded as a “silent pandemic” and a public health issue, especially in low- and middle-income countries (LMICs) like Pakistan^36^. This study elucidates the genetic characteristics of hvKp and cKp isolates recovered from clinical settings. The current study examined *K. pneumoniae* sourced from blood, pus, and urine, comprising several STs. *K. pneumoniae* was most frequently detected in urine, which aligns with the observations reported by others^37^. There has been a significant increase in the prevalence of MDR and Extensively Drug-Resistant (XDR) *K. pneumoniae*, estimated to be over 40% in Pakistan^38^. Notably, carbapenem-resistant *K. pneumoniae* (CRKP) has become more prevalent in Pakistan^39^. Our research supports earlier findings that suggested numerous AMR genes linked to resistance to different medication classes were present in *K. pneumoniae*^6,22,40^. Clinical isolates of *K. pneumoniae* may have more genetic and phenotypic diversity due to the accumulation of various antibiotic resistance genes brought about by horizontal gene transfer^1,16^. The high-risk clone *K. pneumoniae* ST147, known for its association with MDR and pan-drug resistance in chronic infections, was detected in our dataset^41^. In Tuscany, Italy, an epidemic caused by a hypervirulent *K. pneumoniae* ST147 strain carrying a chimeric plasmid harbouring both virulence and resistance genes^42^ was reported, consistent with our findings.

Considering the *bla-SHV* gene has allelic variations in the core genome, all of the isolates in our research were ampicillin-resistant. The two most common SHV variants within them were *bla-SHV-1* and *bla-SHV-11*^43^. Several studies conducted in China have found this variation^44^. Recent investigations have shown the variation *bla-SHV-187* among MDR *Klebsiella* ST29 isolates^45–47^.

Likewise, our analysis revealed the presence of genes such as *bla-CTX-M-15*, *bla-CMH-1*, and *bla-OXA*, linked to beta-lactam resistance. Furthermore, class B (*bla-NDM* gene NDM-1) and class D (*bla-OXA*, including *OXA-48* and *OXA-181*) carbapenemase variants were detected; these variants have been described in isolates from Pakistan^48^. The most prevalent PMQR genes found in our isolates were *qnrS1* and *qepA2*, which is an observation in line with earlier studies (e.g.,^49^). We discovered that quinolone-resistant isolated strains had mutations in both *gyrA* and *parC* genes, similar to those identified in another study^50^. In the *gyrA* gene, among the most common mutations were I83Serine, L83Serine, and D87Asparginine, while the *parC* gene had l80S. The most prevalent aminoglycoside-resistant genes identified in our investigation included *aac(6')-Ib-cr,* and *aac*^3^*-IId*, *armA, strA, strB,* and *rmtB*, which contributed to high-level aminoglycoside resistance^51,52^. The *ermB* and *mphA* genes were found to be associated with resistance to macrolides, lincosamides, and streptogramin B (MLSB) antibiotics^53^. The biocide-resistant gene *qacE-1*, typically present in gram-negative microbes, is widely prevalent in our study. This gene is associated with resistance to sulfonamides, trimethoprim, and aminoglycosides via a multidrug efflux mechanism^54^.

The presence of ESBL genes in comparable genetic environments in isolates of our study and other studies suggests that they promote antibiotic resistance^55^. The insertion sequence *ISEc9* and specific plasmids, particularly those belonging to the IncF family, have been implicated in mediating gene exchange mechanisms that promote the dissemination of ESBL genes^56^. The IncF plasmid, which plays a role in horizontal gene transfer, was identified in our strains^56^. Only the ST859 strain carried an IncC plasmid replicon containing genes conferring resistance to trimethoprim, AmpC β-lactamase, sulfonamides, aminoglycosides, and chloramphenicol^57^. In our study, the ST859 strain carried Col, FIA, and Inc-type plasmids (Inc(L, Q, R, X, Y)), along with the globally disseminated plasmid IncH1B(pNDM), which has previously been reported in Morocco, the United States, and the United Kingdom^57^. The most common sequence type identified in our study was ST147, which exhibited the serotype combination KL64 and O2v1 across all seven strains. *K. pneumoniae* isolates from hospitalised patients have been reported to belong to ST147 in association with the KL64 capsular type^58^. *K.* pneumoniae can be classified into 134 capsular types based on variations in polysaccharide composition, corresponding to different K and O antigens^59^. It is well established that hypervirulent *K. pneumoniae* variants are associated with capsular type K54. This type is particularly linked to sequence type ST29, a hypervirulent strain commonly found within clonal group CG29^60^.

## Conclusion

We present a comprehensive WGS study of *K. pneumoniae* strains from clinical sources in Khyber Pakhtunkhwa, Pakistan, revealing substantial genetic diversity. Notably, three isolates - absent from the TYGS database -harboured key virulence genes and antimicrobial resistance determinants, indicating the presence of rare or underreported variants. The widespread occurrence of virulence and resistance genes highlights their potential contribution to poor therapeutic outcomes and elevated mortality rates. Furthermore, uncommon sequence types, K loci, and spontaneous mutations were detected, emphasising the need for ongoing epidemiological and molecular investigations to inform effective treatment strategies.

## Supplementary Information

Below is the link to the electronic supplementary material.

Supplementary Material 1

## Data Availability

The WGS data generated in this study have been deposited in the NCBI Sequence Read Archive under BioProject accession number PRJNA1148473. The isolates have been deposited in Pathogenwatch, available from https://pathogen.watch/collection/edexwe3w44o9-kleb_pak.
